# Long-term *Staphylococcus aureus* decolonization in patients on home parenteral nutrition: study protocol for a randomized multicenter trial

**DOI:** 10.1186/s13063-018-2732-2

**Published:** 2018-06-28

**Authors:** Michelle Gompelman, Yannick Wouters, Wietske Kievit, Joost Hopman, Heiman F. Wertheim, Chantal P. Bleeker-Rovers, Geert J. A. Wanten

**Affiliations:** 1 Department of Gastroenterology & Hepatology I Infectious Diseases, P.O. Box 9101, 6500 HB Nijmegen, The Netherlands; 2 Department of Gastroenterology and Hepatology, Radboudumc, Nijmegen, The Netherlands; 3 Department for Health Evidence, Radboud University, Nijmegen, The Netherlands; 4 Department Medical Microbiology, Radboudumc, Nijmegen, The Netherlands; 5 Department of Internal Medicine and Infectious Diseases, Radboudumc, Nijmegen, The Netherlands

**Keywords:** Home parenteral nutrition, *Staphylococcus aureus*, Carriage, Long-term, Decolonization

## Abstract

**Background:**

Patients with long-term intestinal failure are usually treated by means of home parenteral nutrition (HPN) where they administer their nutritional formulation intravenously via a central venous access device (mostly a catheter). This implies that such patients are exposed to a lifelong risk of developing *Staphylococcus aureus* bacteremia (SAB). SAB poses a threat to both catheter and patient survival and may lead to frequent hospitalization and a permanent loss of vascular access. In other clinical settings, *S. aureus* carriage eradication has been proven effective in the prevention of *S. aureus* infections. Unfortunately, there is a complete lack of evidence in HPN support on the most effective and safe *S. aureus* decolonization strategy in *S. aureus* carriers. We hypothesized that long-term *S. aureus* decolonization in HPN patients can only be effective if it is aimed at the whole body (nasal and extra-nasal) and is given chronically or repeatedly on indication. Besides this, we believe that *S. aureus* carriage among caregivers, who are in close contact with the patient, are of great importance in the *S. aureus* transmission routes.

**Methods/design:**

The CARRIER trial is a randomized, open-label, multicenter clinical trial in Dutch and Danish hospitals that treat patients on HPN. A total of 138 adult HPN patients carrying *S. aureus* will be randomly assigned to a search and destroy (SD) strategy, a quick and short, systemic antibiotic treatment, or a continuous suppression (CS) strategy, a repeated chronic topical antibiotic treatment. The primary outcome measure is the proportion of patients in whom *S. aureus* is totally eradicated during a 1-year period. Secondary outcomes are time to successful eradication, long-term antimicrobial resistance, adverse events, patient compliance, incidence of (*S. aureus*) infections, catheter removals, mortality rates, *S. aureus* transmission routes, quality of life, and health care costs.

**Discussion:**

The CARRIER trial is designed to identify the most safe and effective long-term *S. aureus* carriage decolonization strategy in HPN patients. This will eventually lead to a better understanding of long-term *S. aureus* decolonization treatments in general. The results of this study will have a great impact on our daily clinical practice, which eventually may result in less *S. aureus-*related infections.

**Trial registration:**

ClinicalTrials.gov; NCT03173053. Registered on 1 June 2017.

**Electronic supplementary material:**

The online version of this article (10.1186/s13063-018-2732-2) contains supplementary material, which is available to authorized users.

## Background

### Intestinal failure and home parenteral nutrition

**Fig. 1:**
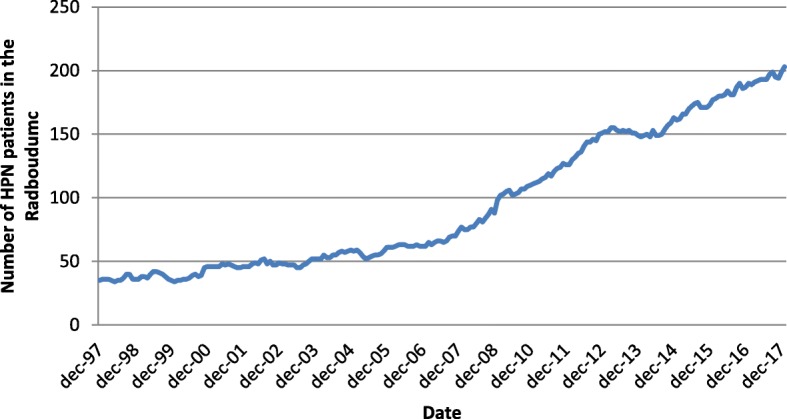
Incidence of patients on home parenteral nutrition (HPN) from 1997 to 2017 in the Radboudumc Nijmegen, The Netherlands. The exponential increase of HPN patients is attributed to a higher patient survival, multimorbidity, and the increase of complex surgical procedures

The number of patients with intestinal failure in the Netherlands has increased exponentially over the past decade, from approximately 100 to 400 patients (Fig. 1). These patients depend on life-long home parenteral (intravenous) nutrition (HPN). HPN is a complex and time-consuming treatment focused around training patients to use their venous catheter and infusion pump at home. With the use of such venous access come frequent life-threatening complications, such as catheter-related bloodstream infections (CRBSIs). The reported incidence of CRBSIs in expert centers ranges from 0.38 to 4.58 episodes per 1000 catheter days and accounts for approximately 70% of all HPN-related hospital admissions [1, 2]. These complications are a threat to both the catheter and patient survival and may lead to permanent loss of vascular access. Because most HPN patients are well-trained to perform complex aseptic techniques to manage their catheters, further improvement of these techniques will likely have no significant impact on infection prevention.

### *Staphylococcus aureus* infections and carriage

*Staphylococcus aureus* related to catheter exit-site, tunnel infections, and bacteremia (SAB) may all lead to complicated infections resulting in catheter loss [1]. On top of this, SABs easily result in secondary metastatic infectious foci leading to high morbidity and mortality rates of 20 to 30% [3–5]. In addition to the psychological impact on patients, healthcare-associated costs are substantial; around $30.000 per infection [5].

By estimation, the prevalence of nasal *S. aureus* carriage in HPN patients is comparable to hemodialysis patients, over 30% [6]. Nasal *S. aureus* carriage is a well-defined risk factor for subsequent *S. aureus* infections, and vice versa. Finally, more than 80% of health care-associated *S. aureus* infections are endogenous of origin [7, 8]. Studies show that the risk of developing *S. aureus* infections is 11.5 times higher among colonized hemodialysis patients compared with uncolonized patients [9]. In addition, approximately 19% of dialysis patients who are *S. aureus* carriers develop a *S. aureus* infection each year [9]. Infection prevention is a key strategy to maintain venous access and to avoid hospitalization. As such, *S. aureus* carriage eradication is instrumental for infection prevention. Besides this, evidence is mounting that *extra-*nasal *S. aureus* colonization is more common than previously believed. The usual decolonization strategies with only mupirocin nasal ointment are probably insufficient because extra-nasal body regions remain colonized with *S. aureus*. Finally, the role of *S. aureus* transmission to the HPN patient by a close partner or caregiver is unclear. Of note, the setting of HPN support poses a unique additional challenge here, since the caregiver is a critical factor in *S. aureus* carriage and transmission because the management of the patient’s venous catheter is often performed by the partner on a continuous basis.

### Rationale for *S. aureus* decolonization in HPN patients

The above demonstrates that evidence-based recommendations for *S. aureus* decolonization are needed, specifically for HPN patients and their caregivers. Unfortunately, current guidelines do not provide such recommendations; patients are currently treated—mostly based on “expert opinion”—with oral antibiotics, topical antibiotics, or nothing at all. This results in large treatment variations between the hospitals and even between clinicians.

We hypothesized that long-term *S. aureus* decolonization in HPN patients can only be effective if the decolonization treatment is aimed at the whole body (nasal and extra-nasal) and is given chronically or repeatedly on indication. The aim of the CARRIER trial is to improve patient care by reliably identifying the most effective and safe long-term *S. aureus* carriage decolonization strategy in HPN patients. Ultimately this will lead to less antimicrobial resistance, less catheter removals, and lower mortality rates. The CARRIER trial protocol was written in accordance with the Standard Protocol Items: Recommendations for Interventional Trials (SPIRIT). The SPIRIT checklist has been included as Additional file 1.

## Methods

### Design

**Fig. 2:**
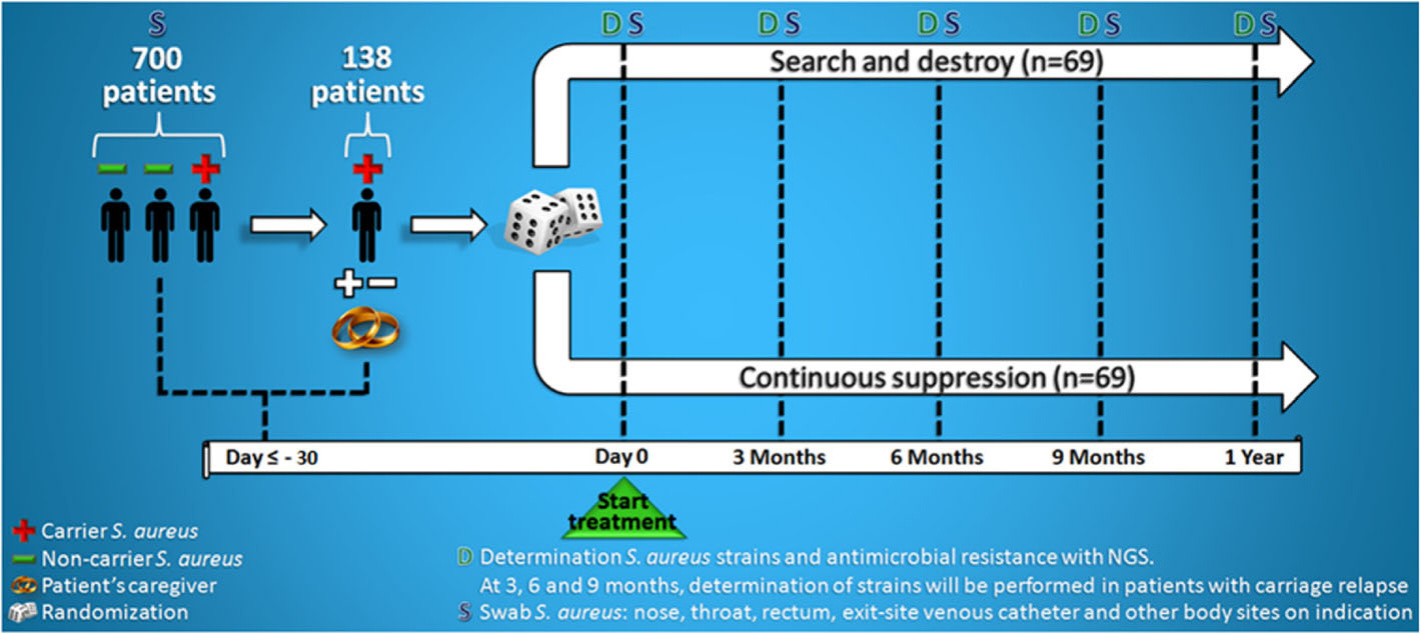
Study design of the CARRIER trial. Seven hundred parenteral nutrition patients will be screened for *S. aureus* colonization. A total of 138 *S. aureus* carriers will be included, of which 69 patients will be randomized to the search and destroy group and 69 patients to the continuous suppression group

**Fig. 3:**
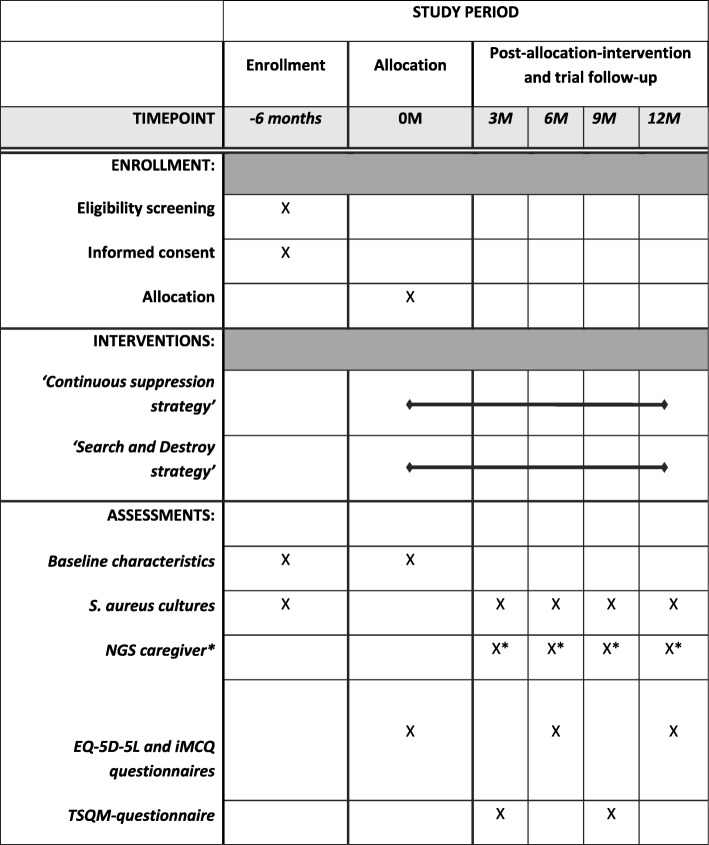
Schedule of enrollment, interventions, and assessments. Schedule of enrollment, interventions, and assessments according to the SPIRIT 2013 guidelines. *NGS* next-generation sequencing, *EQ5D-5 L* EuroQol 5 Dimensions, 5 levels, *TSQM* treatment satisfaction questionnaire measurement, *iMCQ* iMTA Medical cost questionnaire, *M* month(s). *In case of *S. aureus* eradication failure

The CARRIER trial is a multicenter, randomized controlled, open label, superiority trial in adult HPN patients carrying *S. aureus.* It aims to investigate the most effective and safe long-term *S. aureus* carriage decolonization strategy. The trial will recruit 138 patients that will be randomized to either a “search and destroy” (SD) group or a “continuous suppression” (CS) group (Figs. 2 and 3). The study design mirrors the real-life setting with respect to costs and effects.

#### Setting

The setting of this study is the outpatient clinic of the Endocrinology and Metabolism department of one Dutch academic hospital and the Gastroenterology and Hepatology departments of one Dutch and two Danish academic hospitals. The expected duration of this study is 3 years (6 months of preparation time and 2.5 years for enrollment and follow-up). The Danish HPN population is very similar to the Dutch HPN population in terms of infection rates, methicillin-resistant *S. aureus* (MRSA) prevalence, provided healthcare, healthcare system, and (benign) diseases leading to intestinal failure.

#### Trial population

All adult patients with intestinal failure on HPN will be screened for *S. aureus* carriage. With the expectation that at least 30% of the HPN patients are *S. aureus* carriers, approximately 700 patients will need to be screened. In case *S. aureus* carriage is confirmed, the patient will be screened for further eligibility and asked to enroll in the trial.

#### Inclusion criteria

In order to be eligible to participate in this study, a patient must meet all of the following criteria:

- Patient is fully able to understand the nature of the proposed intervention.
- Patient is diagnosed with intestinal failure and receives HPN.
- Written informed consent is provided by the patient before entering the trial.
- Age ≥ 18 years.
- Estimated life expectancy ≥ 1 year.
- Patient colonized with *S. aureus* (nasal and/or extra-nasal).

#### Exclusion criteria

A patient who meets any of the following criteria will be excluded from participation:

- Cannot be expected to comply with the trial plan (substance abuse, mental condition).
- Pregnant or breastfeeding women.
- Continuous exposure to MRSA (e.g., pig farmer).
- Allergy for both chlorhexidine and/or betadine.
- No options for any of the study drugs (systemic and/or topical antibiotics) due to resistance, allergies and/or interacting co-medication.
- Active *S. aureus* infection.
- Currently on treatment with antibiotics active against *S. aureus*.
- Decolonization (including mupirocin) treatment in the previous 2 months.
- The presence of an irremovable nasal foreign body.
- Aspartate transaminase (AST) and alanine transaminase (ALT) levels more than five times the upper limit of normal or liver failure.

### Study intervention

Prior to the start of the trial, HPN patients have been screened for *S. aureus* carriage with swabs of the nose, throat, rectum, exit-site catheter, and body regions on indication (e.g. stoma, wound, skin lesion). Subsequently, enrolled patients will visit the regular outpatient clinic appointments every 6 months, in accordance with current guidelines [10].

**Fig. 4:**
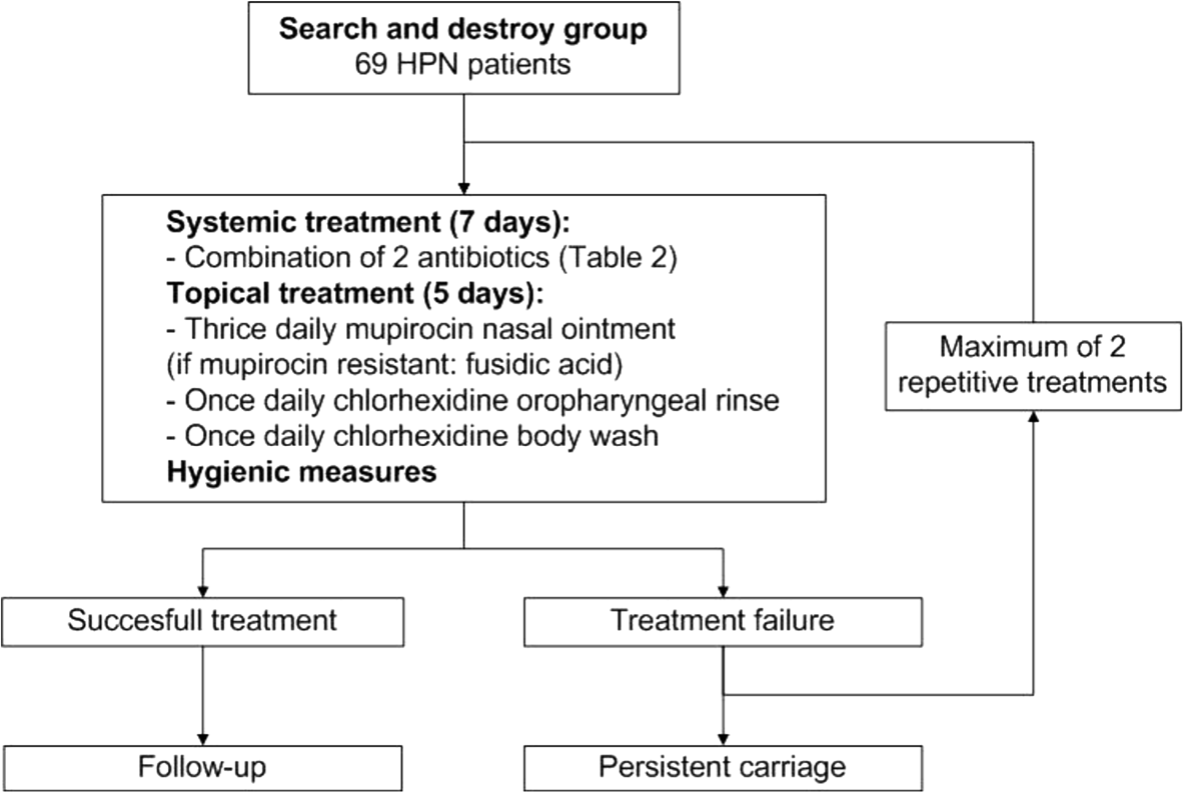
Search and destroy group strategy. Flow chart of the search and destroy group treatment strategy

**Fig. 5:**
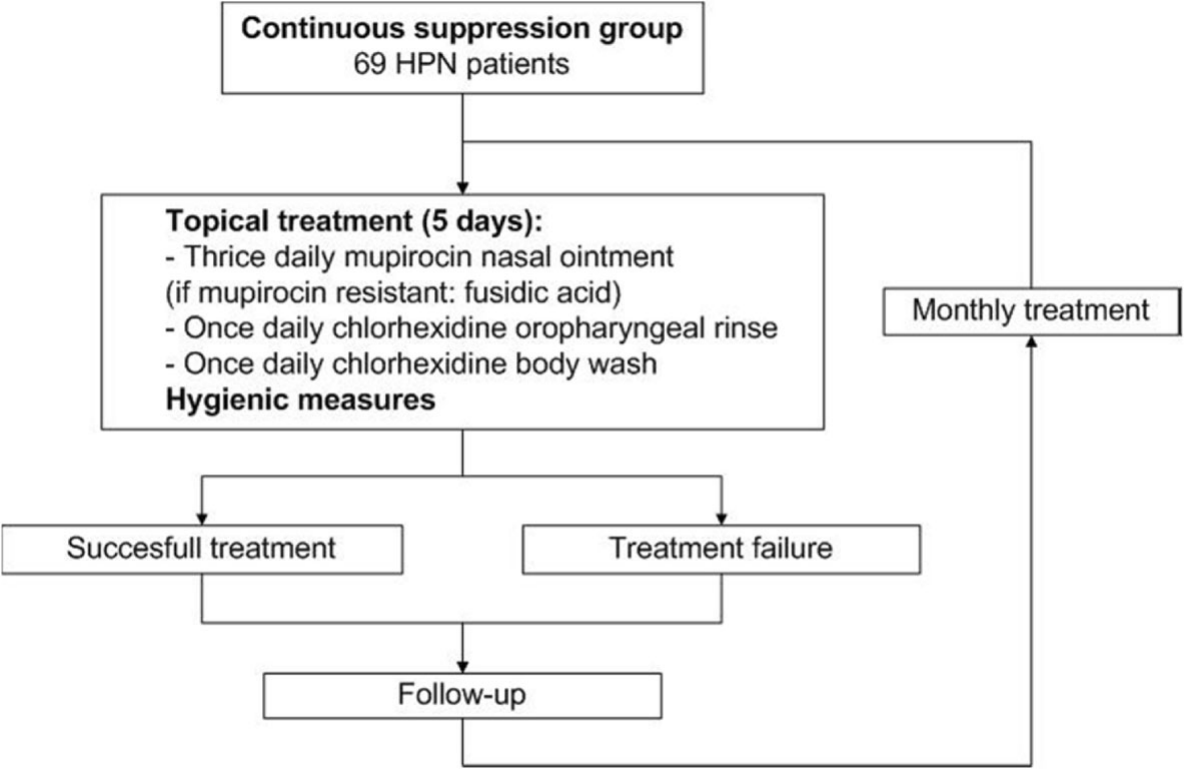
Continuous suppression group strategy. Flow chart of the continuous suppression group treatment strategy

After randomization, patients will be allocated to one of the following two treatment strategies:

- *Search and destroy (SD) strategy* focuses on the quick and short, systemic antibiotic eradication of *S. aureus* (Fig. 4)*.* Patients will receive treatment with mupirocin nasal ointment, a chlorhexidine oropharyngeal rinse and body wash, and two systemic antibiotics for one week, according to the Dutch MRSA guideline [10]. Hygienic measures consist of wearing clean clothing every day and frequent change of towels and bed clothes during treatment. Combination therapy is indicated because of improved effectiveness and a decreased chance of developing resistance. After one cycle of treatment, a set of body swabs (nose, throat, rectum, exit-site catheter, and body regions on indication (e.g. stoma, wound, skin lesion) will be taken weekly for 3 weeks to confirm total eradication. In case of persistent *S. aureus* carriage, patients will be treated again. Patients will not receive more than three treatment cycles per round. A successful treatment is defined as three consecutive negative sets of surveillance cultures obtained at least 48 h after completion of treatment and distributed over a period of at least 14 days. Treatment failure is defined when a patient is still a *S. aureus* carrier after three treatment cycles. Relapse of *S. aureus* carriage will be subsequently monitored at the standard new swab rounds, every 3 months. In case of relapse, patients will be treated again as well.
- *Continuous suppression (CS) strategy* focuses on the continuous, topical eradication o*f S. aureus* (Fig. 5)*.* Whether or not patients are carriers during the year, they will receive mupirocin nasal ointment, an oropharyngeal chlorhexidine rinse, and body wash for extra-nasal body regions every month for 5 days. The same hygienic measures as in the SD strategy group will be applied. Standard swabs of the nose, throat, rectum, exit-site catheter, and other body regions on indication will be taken every 3 months to monitor full body eradication.

### Outcome measures

- *Primary outcome*
  Proportion of patients in whom *S. aureus* is totally eradicated during a 1-year time period.
  Total eradication is defined as 100% of all swabs (nose, throat, rectum, exit-site catheter, and body regions on indication) being negative for *S. aureus* per measured time point.
- *Secondary outcomes*
  Secondary outcomes are listed in Table 1.

**Table 1:** Secondary outcome measures

Secondary outcome measure	Data collection instrument or method
Incidence of *S. aureus* infections	(S)AE forms every 3 months
Overall incidence of infections	(S)AE forms every 3 months
Mortality	Data from electronic patient file
Long-term antimicrobial resistance	Culture results every 6 months and NGS
Number of catheter removals	Data from electronic patient file
Time to first catheter-related infection	(S)AE forms every 3 months and data from electronic patient file
Successful *S. aureus* eradication per body site	Culture results
Relapse rate of *S. aureus* carriage	Culture results and NGS
Transmission routes	Caregivers culture results and NGS
*S. aureus* transmission routes	NGS
Adverse events	(S)AE forms every 3 months
Predictors for infections and treatment failure or success	Binominal regression analysis
Patient compliance	Medication files, counting pills, trial-specific medication diary, modified Morisky Medication adherence questionnaire
Generic health related quality of life	EuroQol 5 dimension, 5 levels questionnaire (EQ5D-5 L)
Treatment satisfaction	TSQM vII questionnaire
Healthcare related costs	iMCQ questionnaire

*iMCQ* iMTA Medical Consumption Questionnaire, *NGS* next-generation sequencing, *(S)AE* (serious) adverse event, *TSQM* Treatment Satisfaction Questionnaire for Medication

### Procedures, participants, and analyses

#### Recruitment and screening

The subjects of this study will be identified from local HPN databases of the different study sites. Patients will be selected by the attending physicians and local nurse practitioners. The local investigator will provide information about the study by phone and will subsequently send information material, including the Informed Consent Agreement, to the patient’s home address. Informed consent will be obtained before the start of any study-specific procedures (e.g., randomization). Data will be saved in a documentation system based on electronic Case Report Forms (eCRFs; CastorEDC 2017.9). CastorEDC is compliant with ICH-GCP and all the regulations required by FDA 21CFR Part 11 for electronic data management.

#### Randomization

Randomization will be performed centrally by the principal investigators using an online randomization module (CASTOR EDC 2017.9) in a 1:1 ratio to either a SD group or a CS group (Figs. 2 and 3). Randomization will be stratified by country to balance differences in national treatment guidelines and different antimicrobial susceptibility patterns. Variable block randomization consisting of two, three, or four marbles will be used to provide treatment allocation in equal proportions. We considered a blinded design, but the treatment strategy protocols of both groups differ to such an extent that this precludes adequate blinding. Besides this, an unblinded (more pragmatic) design fits best with the current ideas about the external validity of cost-effectiveness studies.

#### Baseline examination

At the start of the trial, patient characteristics (from existing electronic patient system, medical history, and/or by physical examination), results of earlier obtained culture swabs, and/or blood results will be collected on the eCRF by the investigator.

#### Choice of antibiotic drug(s)

Patients will be treated according to the respective marketing authorizations and national MRSA guidelines as previously mentioned (Table 2). The choice of antibiotic drugs depends on known allergies, expected decreased absorption in the case of short bowel, and susceptibility patterns of the *S. aureus* isolates. If during the course of the study the treating physician or consulted specialist infectious diseases feels that a particular antibiotic drug is clearly indicated or contra-indicated in a patient, the choice of the antibiotic drug may be changed at his/her discretion.

**Table 2:** Systemic combination therapy for eradication of MRSA carriage in complicated carriage according to the Dutch MRSA guideline [10]

Dutch MRSA guideline	Antibiotic 1	Antibiotic 2
Recommended	Sulfamethoxazole/Trimethoprim 960 mg twice daily *or* Doxycycline 200 mg once daily	Rifampicin 600 mg twice daily
Alternative^ɑ^	Clindamycin 600 mg thrice daily *or* Clarithromycin (or another macrolide) 500 mg twice daily *or* Ciprofloxacin (or another quinolone) 750 mg twice daily *or* Fusidic acid 500 mg thrice daily *or* Linezolid 600 mg twice daily	Fusidic acid 500 mg thrice daily

All treatments are prescribed preferably by means of tablets or capsules. The dosages in this table are the recommended dosages for an adult patient of about 70 kg. Combination therapy will be used because of better effectiveness and a decreased chance of developing resistance

^ɑ^Alternative options should only be used when there is a contraindication (e.g., in vitro resistance, intolerance) for the recommended options

#### Caregivers

In case of treatment failure, a patient’s caregiver (often the patient’s partner) will be asked to participate in the trial to investigate transmission routes from the caregiver to the patient. Presence of a caregiver is not mandatory for the patient’s participation. Caregivers will not be randomized nor treated. Next-generation sequencing (NGS) will be used to determine the type of *S. aureus* strains and to compare them with the strains retrieved from caregivers and/or previous *S. aureus* isolates.

#### Bacteriological methods

##### Identification of *S. aureus* and storage of positive cultures

To screen patients for *S. aureus* carriage, flocked swabs with moisture (Eswab) will be used. To ensure high quality of this procedure, training for the nurses and patients’ caregivers and an instruction brochure will be provided prior to the start of the study. After collection, the swab will be placed in 100 μl of saline and centrifuged. Real-time PCR (polymerase chain reaction) and conventional cultures will be used for the detection of *S. aureus* isolates. Cultures will be performed after broth enrichment, which improves the performance of chromogenic solid media for the detection of *S. aureus* in clinical samples (sensitivity of 95–97%). This is according to the national MRSA detection guidelines and will be the same for all the participating centers [11]. For perineum swabs, CAN agar plates will be used. This is a selective microbiological medium for *S. aureus*, which inhibits Gram-negative bacteria during culture. All *S. aureus* isolates will be stored at − 80 °C in glycerol-containing liquid media for the duration of 1 year after the end of the study.

##### Next-generation sequencing

NGS, performed with Illumina NextSeq (Illumina, San Diego, CA, USA), will be used in a subgroup of subjects (for example, in case of relapse or occurrence of active *S. aureus* infection). *S. aureus* strains will be distinguished by comparing single nucleotide polymorphisms (SNPs) in the core genome and by relating these SNPs to the genealogical tree of *S. aureus*. A cut-off point of 30 different SNPs will be used to classify *S. aureus* strains as distinct [12].

#### Follow-up

Patients will be followed for 1 year. At months 6 and 12, swabs will be obtained at the outpatient clinic and questionnaires regarding adverse events (AEs) will be collected. In between outpatient visits, at 3 and 9 months, swabs will be sent to the laboratory and patients will be interviewed via telephone to assess adverse events and treatment satisfaction with a validated questionnaire (TSQM vII). At inclusion and 6 and 12 months, generic health-related quality of life and health care costs will be measured with questionnaires as well (EQ-5D-5 L, iMCQ). Discontinuation of study treatment by patients will be recorded, including the reason for discontinuation. If possible, a final visit procedure (collecting swabs 1 year after inclusion) will be performed.

#### Patient compliance

At months 6 and 12, patient compliance will be monitored by medication files, a trial-specific medication diary (on paper or digital via medication application MedApp), and specific questions regarding medication adherence. In addition, all patients will have a study-specific list with information about their *S. aureus* screening and treatment protocol. We chose this method for measuring adherence because it is pragmatic and reliable, without influencing the daily practice too much.

#### Data management

All data will be handled confidentially and, where necessary, anonymized. The investigator will record all data in an eCRF. All files will be encrypted by a password only known by the investigators. Originals of laboratory or other tests related to the study will be kept on file at the study site. This study will be performed in accordance with the legal laws formulated in the Dutch “Wet Bescherming Persoonsgegevens”.

### Analysis

#### Sample size and power calculation

Hardly any literature is available regarding long-term efficacy of the CS group on total *S. aureus* carriage eradication. Even when articles describe similar eradication strategies as the CS group, endpoints vary from only local, nasal *S. aureus* eradication to *S. aureus* infections. Consequently, total body site (de)colonization has not been described thoroughly. Based on a few studies with quite similar eradication strategies, it is, however, possible to make an assumption about the long-term efficacy [10, 13–16]. The eradication rate of the SD strategy group is expected to be 77% and for the CS strategy group 55% over 1 year. Based on the settings binary outcome (carriage yes/no), superiority trial, power (1 − β) of 80% and a significance level (α) of 5%, a total of 138 patients (2 × 69) are required to detect a significant increase from 55 to 77%.

#### Statistical analysis

The primary analysis will be based on the intention-to-treat principle.

##### Primary study parameter(s)

The primary endpoint (proportion of *S. aureus* eradication during a 1-year time period, using logistic mixed models) will be expressed in terms of odds ratios and 95% confidence intervals (CI). If the 95% CI does not contain value 1, we can conclude that either of the strategies is significantly different from the other.

We choose a logistic mixed-effect model because it is a linear model that fits best for this longitudinal study using repeated dichotomous measures. In addition, logistic mixed-effects models have a better distinguishing ability. It is expected that this results in an even greater power than the more simplistic area under the curve model that was used for the power analysis.

##### Secondary study parameter(s)

Continuous data such as patient characteristics (age, sex, venous access, CRBSIs) will be presented as means ± standard deviation and in case of skewed distributions as medians and range. Dichotomous outcomes will be summarized as percentages (N events/N total). Parameters will be compared between both groups by the Student’s *t*-test, Wilcoxon rank sum test, Chi2-test or Fischer exact test depending on the type of outcome and distribution. A two-tailed *p* value < 0.05 is considered statistically significant. No corrections for multiple tests are applied. Known predictors (such as socio-economic status, pet ownership, location of *S. aureus* carriage, *S. aureus* carriage by caregiver, and active skin disease) for infections and treatment failure/success will be analyzed using a logistic and/or linear regression model.

##### Cost-effectiveness analysis

The cost-effectiveness gain related to the proposed eradication strategies lies in the comparison with having no strategy at all (doing nothing) and the prevention of infections. “Doing nothing” is not included in this trial because it is considered unethical as we clearly expect no eradication at all. The number of infections is expected to be low in this trial and the positive effects of prevention of infection do extend the time horizon of this trial. Therefore, the prospectively gathered data in this trial will be combined with literature into a Markov model in order to estimate the incremental cost-effectiveness (ICER) from a health care perspective over a time horizon of 5 years. The model is needed to estimate the infection rate in a doing-nothing strategy, to relate infections to quality of life losses, and to extrapolate the trial data. During the trial the quality of life of patients will be assessed by the EQ-5D-5 L at baseline and 6 and 12 months and the result (Dutch tariff) will be used to derive a QALY estimate for each patient according to the trapezium rule. The cost analysis consists of two main parts. First, at the patient level, volumes of care related to the HPN care and infections will be measured by means of the iMTA Medical Consumption Questionnaire (iMCQ). In addition, the medication use will be derived from the electronic patient records. Loss of productivity due to illness or recovery will not be included in the analyses because, on estimation, only 5% of the HPN patients perform paid (or unpaid) labor.

The second part of the cost analysis consists of determining the cost prices for each volume of consumption. The standard cost prices from the “Dutch Guidelines for Cost Analyses” and https://www.medicijnkosten.nl/ will be used. In the end, volumes of care will be multiplied by the cost prices for each volume of care to calculate costs. Two incremental cost-utility ratio’s (ICURs), expressed as costs per QALY gained, will be calculated—one for each strategy compared to doing nothing. Model-based cost-effectiveness analyses will be carried out using probabilistic sensitivity analysis, by Monte Carlo simulation, to take all uncertainties surrounding the input parameters into account. The costs will be included across a gamma distribution, the probabilities and utilities with beta distributions, which is according to the guidelines of the ISPOR. The results of the 5000 simulations will be plotted in cost-effectiveness planes and in willingness to pay curves.

##### Budget impact analysis

A budget impact analysis (BIA) will be performed according to the ISPOR principles of Good Practice for BIA. This BIA allows prediction of the financial consequences related to the adaption and implementation of one of the eradication strategies, in order to determine the affordability of the intervention. Data will be used that reflect the size and characteristics of the HPN population in the Netherlands together with the results of this trial (effect sizes, resource use, etc). When relevant, budget impact analyses are generated as a series of scenario analyses.

### Quality and safety

In general, this study aims to restrict the physical and mental burdens for the subject as much as possible. The physical risks that are introduced by this study to the participants are believed to be minimal. The risk derived from collecting the culture swabs is negligible if performed by well trained physicians, nurse practitioners, and/or the patient’s own caregiver. For this, we will provide an information brochure and give instructions during the outpatient clinic visit. Next to this, the systemic antibiotics as prescribed in the SD group have the potential risks of causing side effects, certain toxicities and allergies, or intolerance. These potential risks, however, are all well known because the antibiotics that are prescribed are part of the routine care and are prescribed following the current national guidelines. During the study sufficient medical health assistance (nurse practitioners, attending physician or principle investigator) will be present at all times in the hospital and reachable by phone to cope with unexpected events. Serious adverse events will be reported through a web portal to the central committee on research involving human subjects (in Dutch: Centrale Commissie Mensgebonden Onderzoek) and the accredited institutional review board (https://www.toetsingonline.nl/). The remaining events are recorded in an electronic database and reviewed annually by an independent monitor.

### Monitoring

During the trial, two independent monitors (one in the Netherlands and one in Denmark) will visit the sites yearly to check the completeness of patient records, the accuracy of entries on the CRFs, the adherence to the protocol and to Good Clinical Practice (GCP), the progress of enrollment, and also to ensure that trial devices are being stored, dispensed, and accounted for according to specifications.

## Discussion

This trial will provide guidance for further policy development and implementation of (long-term) *S. aureus* decolonization protocols and other novel infection/transmission preventive strategies. HPN patients, and likely other chronic patient groups such as hemodialysis patients, will benefit from an evidence-based effective and safe long-term *S. aureus* decolonization protocol. Ultimately such a protocol will lower *S. aureus* infections and reduce subsequent hospitalizations, catheter removals, mortality, antimicrobial resistance, and costs.

We believe that with this project we can gain essential knowledge that fills a significant gap in our current understanding of (long-term) *S. aureus* decolonization treatments. As such, the CARRIER trial will prove the efficacy of a long-term *S. aureus* decolonization strategy in HPN patients for the first time. The study will give insight into other important outcome measures such as antimicrobial resistance, *S. aureus* transmission, patient compliance and—as a completely new feature—the role of the caregiver and his/her carrier status.

During the trial, antibiotic drugs will be used according to the national MRSA eradication guidelines [10]. These drugs are also used in clinical practice for eradication of methicillin-sensitive *S. aureus* (MSSA) since there is no separate guideline for MSSA eradication. The MRSA guideline based their advice on studies that were not restricted to the eradication of MRSA only, but included studies evaluating MSSA eradication as well. There is no evidence that any of the treatment options included in the MRSA guideline have a more favorable risk:benefit ratio in the HPN population. Allowance of different treatment regimens that are included in the MRSA guideline enables us to include patients with certain allergies or potential interacting co-medication as well. This is in line with current clinical practice and enables us to include enough patients to answer the research question.

From the patients’ perspective, the trial will have many advantages as well. Implementation of the trial results might lead to less morbidity (less hospitalizations and complications such as *S. aureus* infections and catheter removals) and eventually a lower mortality. In addition, we think that the results of this study will most likely have an impact on daily clinical practice, since our aim is to implement the findings in the (inter)national guidelines on chronic intestinal failure in adults.

Strengths of this trial include its multicenter, randomized controlled design in different international centers, which will increase the (international) generalizability of the study. Besides this, barely any studies have investigated effective long-term *S. aureus* eradication strategies. The outcomes include mostly objective measures to determine successful *S. aureus* eradication: the results of bacterial cultures and the additional whole-genome sequencing in some cases. The questionnaires used for the subjective patient-reported outcomes (e.g., impact on overall quality of life (QOL), treatment satisfaction, and health care related costs) are well-accepted, reliable, and mostly validated (TSQM vII, EQ-5D-5 L and iMCQ). Lastly, the burden for patients is expected to be minimal since the feasible treatment protocol chosen has limited impact on daily life.

In summary, the findings of this study will help to determine what the most effective and safe long-term *S. aureus* decolonization strategy in HPN patients is. The trial will determine the relative contribution of two different decolonization strategies on successful eradication, reduction of catheter-related bloodstream infections (CRBSIs), and eventually a lower mortality.

## Trial status

Currently, the trial is ongoing and patient recruitment started at the end of 2017. The estimated end date will be in Q4 of 2019.

## Additional file

Additional file 1: Standard Protocol Items: Recommendations for Interventional Trials (SPIRIT) Checklist. (DOC 122 kb)

## Abbreviations

AE: Adverse event
ANOVA: Analysis of variance
BIA: Budget impact analysis
CRBSI: Catheter-related bloodstream infection
CS: Continuous suppression
EQ5D-5 L: EuroQol 5 dimensions, 5 levels
HPN: Home parenteral nutrition
ICUR: Incremental cost-utility ratio
iMCQ: iMTA Medical Consumption Questionnaire
ISPOR: International Society for Pharmacoeconomics and Outcomes Research
MRSA: Methicillin-resistant *Staphylococcus aureus*
MSSA: Methicillin-sensitive *Staphylococcus aureus*
NGS: Next-generation sequencing
QALY: Quality-adjusted life year
QOL: Quality of life
SAB: *S. aureus* bacteremia
SAE: Serious adverse event
SD: Search and destroy
SNP: Single nucleotide polymorphism
SWAB: Stichting Werkgroep AntibioticaBeleid
TSQM: Treatment Satisfaction Questionnaire for Medication
